# Metric projection for dynamic multiplex networks

**DOI:** 10.1016/j.heliyon.2016.e00136

**Published:** 2016-08-04

**Authors:** Giuseppe Jurman

**Affiliations:** Fondazione Bruno Kessler, Trento, Italy

**Keywords:** Information science, Computational mathematics, Applied mathematics, Computer science

## Abstract

Evolving multiplex networks are a powerful model for representing the dynamics along time of different phenomena, such as social networks, power grids, biological pathways. However, exploring the structure of the multiplex network time series is still an open problem. Here we propose a two-step strategy to tackle this problem based on the concept of distance (metric) between networks. Given a multiplex graph, first a network of networks is built for each time step, and then a real valued time series is obtained by the sequence of (simple) networks by evaluating the distance from the first element of the series. The effectiveness of this approach in detecting the occurring changes along the original time series is shown on a synthetic example first, and then on the Gulf dataset of political events.

## 1 Introduction

When the links connecting a set of *N* nodes arise from *k* different sources, a possible representation for the corresponding graph is the construction of *k* networks on the same *N* nodes, one for each source. The resulting structure is known as a multiplex network, and each of the composing graphs is called a layer. Multiplex networks are quite effective in representing many different real-world situations [1], [2], [3], and their structure helps extracting crucial information about the complex systems under investigation that would instead remain hidden when analyzing individual layers separately [4], [5], [6]; furthermore, their relation with time series analysis techniques has recently gained interest in the literature [7]. A key property to be highlighted is the correlated multiplexity, as stated in [8]: in real-world systems, the relation between layers is not at all random; in fact, in many cases, the layers are mutually correlated. Moreover, the communities induced on different layers tend to overlap across layers, thus generating interesting mesoscale structures.

These observations guided the authors of [9] in defining a network having the layers of the original multiplex graph as nodes, and using information theory to define a similarity measure between the layers themselves, so to investigate the mesoscopic modularity of the multiplex network. Here we propose to pursue a similar strategy for defining a network of networks derived from a multiplex graph, although in a different context and with a different aim. In particular, we project a time series of multiplex networks into a series of simple networks to be used in the analysis of the dynamics of the original multiplex series. The projection map defining the similarity measure between layers is induced by the Hamming–Ipsen–Mikhailov (HIM) network distance [10], a glocal metric combining the Hamming and the Ipsen–Mikhailov distances, used in different scientific areas [11], [12], [13], [14], [15], [16]. The main goal in using this representation is the analysis of the dynamics of the original time series through the investigation of the trend of the projected evolving networks, by extracting the corresponding real-valued time series obtained computing the HIM distance between any element in the series and the first one.

For instance, we show on a synthetic example that this strategy is more informative than considering statistics of the time series for each layer of the multiplex networks, or than studying the networks derived collapsing all layers into one including all links, as in [17], [18] when the aim is detecting the timesteps where more relevant changes occur and the system is undergoing a state transition (tipping point) or it is approaching it (early warning signals). This is a classical problem in time series analysis, and very diverse solutions have appeared in literature (see [19] for a recent example). Here we use two different evaluating strategies, the former based on the fluctuations of mean and variance [20] (implemented in the R package *changepoint* https://cran.r-project.org/web/packages/changepoint/index.html), and the latter involving the study of increment entropy indicator [21].

We conclude with the analysis of the well known Gulf Dataset (part of the Penn State Event Data) concerning the 304.401 political events (of 66 different categories) occurring between 202 countries in the 10 years between 15 April 1979 to 31 March 1991, focusing on the situation in the Gulf region and the Arabian peninsula. A major task in the analysis of the Gulf dataset is the assessment of the translation of the geopolitical events into fluctuations of measurable indicators. A similar network-based mining of sociopolitical relations, but with a probabilistic approach, can be found in [22], [23], [24]. Here we show the effectiveness of the newly introduced methodology in associating relevant political events and periods to characteristic behaviors in the dynamics of the time series of the induced networks of networks, together with a simple overview of the corresponding mesoscale modular structure.

## 2 Background

**Figure 1:**
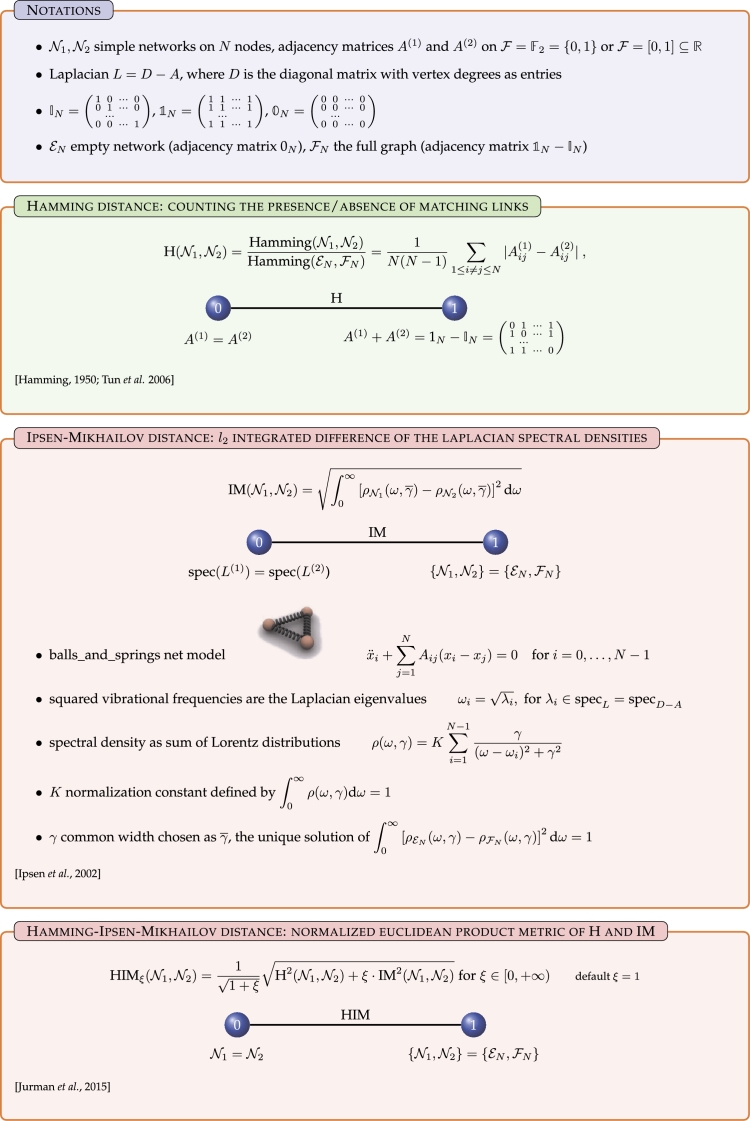
Summary of the definitions of the HIM distance and its Hamming (H) and Ipsen–Mikhailov (IM) components.

The Hamming–Ipsen–Mikhailov (HIM) metric [10], [25] is a distance function quantifying in the real interval [0,1] the difference between two networks on shared nodes. The HIM metric linearly combines an edit distance, the Hamming (H) [26], [27], [28] and a spectral distance, the Ipsen–Mikhailov (IM) [29]. Edit distances are local metrics, functions of insertion and deletion of matching links, while spectral measures are global distances, functions of the network spectrum. Local functions disregards the overall network structure, while spectral measures cannot distinguish isospectral graphs. As its characterizing feature, HIM is a glocal distance that overcomes the drawbacks of local and global metrics when separately considered. Furthermore, its definition can be naturally extended to directed networks. Hereafter we give a brief description of the H, IM and HIM distances, graphically summarized in Figure 1.

*Notations*. Let $N_{1}$ and $N_{2}$ be two simple networks on *N* nodes, whose adjacency matrices are $A^{\left(1\right)}$ and $A^{\left(2\right)}$, with $a_{ij}^{\left(1\right)},a_{ij}^{\left(2\right)}\in F$, where $F=F_{2}=\{0,1\}$ for unweighted graphs and F=[0,1]⊆R for weighted networks. Let then $I_{N}$ be the N×N identity matrix $I_{N}=\left(\begin{array}{cccc} 1 & 0 & \cdots & 0\\ 0 & 1 & \cdots & 0\\ & \cdots \\ 0 & 0 & \cdots & 1 \end{array}\right)$, let $1_{N}$ be the N×N unitary matrix with all entries equal to one and let $0_{N}$ be the N×N null matrix with all entries equal to zero. Denote then by $E_{N}$ the empty network with *N* nodes and no links (with adjacency matrix $0_{N}$) and by $F_{N}$ the clique (undirected simple full network) with *N* nodes and all possible N(N−1) links, whose adjacency matrix is $1_{N}-I_{N}$. Finally, the Laplacian matrix *L* of an undirected network is defined as the difference L=D−A between the degree matrix *D* and the adjacency matrix *A*, where *D* is the diagonal matrix of vertex degrees. *L* is positive semidefinite and singular, with eigenvalues $0=\lambda_{0}\leq \lambda_{1}\leq \cdots \leq \lambda_{N-1}$.

### 2.1 The Hamming distance

The Hamming distance, one of the most common dissimilarity measures in coding and string theory and recently used also for network comparison, evaluates the presence/absence of matching links on the two compared networks. In terms of adjacency matrices, the expression for the normalized Hamming metric *H* reads as

$$\begin{array}{ccc} H(N_{1},N_{2}) & = & \frac{\text{Hamming}(N_{1},N_{2})}{\text{Hamming}(E_{N},F_{N})}=\frac{\text{Hamming}(N_{1},N_{2})}{N(N-1)}\\ & = & \frac{1}{N(N-1)}\sum_{1\leq i\neq j\leq N}|A_{ij}^{\left(1\right)}-A_{ij}^{\left(2\right)}|\;, \end{array}$$

where the normalization factor N(N−1) bounds the range of the function H in the interval [0,1]. The lower bound 0 is attained only for identical networks $A^{\left(1\right)}=A^{\left(2\right)}$, the upper limit 1 for complementary networks $A^{\left(1\right)}+A^{\left(2\right)}=1_{N}-I_{N}$. When $N_{1}$ and $N_{2}$ are unweighted networks, $H(N_{1},N_{2})$ is just the fraction of different matching links over the total number N(N−1) of possible links between the two graphs.

### 2.2 The Ipsen–Mikhailov distance

The Ipsen–Mikhailov IM metric stems from the realization of an *N* nodes network as an *N* molecules system M connected by identical elastic springs, according to the adjacency matrix *A*. The dynamics of the spring-mass system M can be described by the set of *N* differential equations

$${\overset{¨}{x}}_{i}+\sum_{j=1}^{N}A_{ij}(x_{i}-x_{j})=0\;\text{for}\;i=0,\ldots ,N-1\;.$$

The vibrational frequencies of M are given by $\omega_{i}=\sqrt{\lambda_{i}}$, while the spectral density for a graph in terms of the sum of Lorentz distributions is defined as

$$\rho (\omega ,\gamma )=K\sum_{i=1}^{N-1}\frac{\gamma }{{\left(\omega -\omega_{i}\right)}^{2}+\gamma^{2}}\;,$$

where *γ* is the common width and *K* is the normalization constant defined by the condition $\int_{0}^{\infty }\rho (\omega ,\gamma )d\omega =1$, and thus

$$K=\frac{1}{\gamma \sum_{i=1}^{N-1}\int_{0}^{\infty }\frac{d\omega }{{\left(\omega -\omega_{i}\right)}^{2}+\gamma^{2}}}\;.$$

The scale parameter *γ* specifies the half-width at half-maximum, which is equal to half the interquartile range. Then the spectral distance $\epsilon_{\gamma }$ between two graphs $N_{1}$ and $N_{2}$ on *N* nodes with densities $\rho_{N_{1}}(\omega ,\gamma )$ and $\rho_{N_{2}}(\omega ,\gamma )$ can be defined as

$$\epsilon_{\gamma }(N_{1},N_{2})=\sqrt{\int_{0}^{\infty }{\left[\rho_{N_{1}}(\omega ,\gamma )-\rho_{N_{2}}(\omega ,\gamma )\right]}^{2}d\omega }\;.$$

Since $\arg\max_{\left(N_{1},N_{2}\right)}\epsilon_{\gamma }(N_{1},N_{2})=(E_{N},F_{N})$ for each *N*, denoting by $\bar{\gamma }$ the unique solution of $\epsilon_{\gamma }(E_{N},F_{N})=1$, the normalized Ipsen–Mikhailov distance between two undirected networks can be defined as

$$\mathrm{IM}(N_{1},N_{2})=\epsilon_{\bar{\gamma }}(N_{1},N_{2})=\sqrt{\int_{0}^{\infty }{\left[\rho_{N_{1}}(\omega ,\bar{\gamma })-\rho_{N_{2}}(\omega ,\bar{\gamma })\right]}^{2}d\omega }\;,$$

so that IM is bounded between 0 and 1, with upper bound attained only for $\left\{N_{1},N_{2}\}=\{E_{N},F_{N}\right\}$.

### 2.3 The Hamming–Ipsen–Mikhailov distance

Consider now the cartesian product *Z* of two metric spaces (N(N),H) and (N(N),IM), where N(N) is the set of all simple undirected networks on *N* nodes endowed either with the Hamming metric H or with the Ipsen–Mikhailov distance IM. Define then on *Z* the one-parameter Hamming–Ipsen–Mikhailov distance HIM as the $L_{2}$ (Euclidean) product metric of H and $\sqrt{\xi }\cdot$ IM, normalized by the factor $\frac{1}{\sqrt{1+\xi }}$, for ξ∈[0,+∞):

$$\begin{array}{cc} {\mathrm{HIM}}_{\xi }(N_{1},N_{2}) & =\frac{1}{\sqrt{1+\xi }}{\left\|(H(N_{1},N_{2}),\sqrt{\xi }\cdot \mathrm{IM}(N_{1},N_{2}))\right\|}_{2}\\ & =\frac{1}{\sqrt{1+\xi }}\sqrt{H^{2}(N_{1},N_{2})+\xi \cdot {\mathrm{IM}}^{2}(N_{1},N_{2})}\;, \end{array}$$

where in what follows we will omit the subscript *ξ* when it is equal to one. Note that, apart from extreme values, the qualitative impact of *ξ* is minimal in practice, and in what follows ξ=1 will always be assumed. The metric ${\mathrm{HIM}}_{\xi }(N_{1},N_{2})$ is bounded in the interval [0,1], with the lower bound attained for every couple of identical networks, and the upper one attained only on the pair $\left(E_{N},F_{N}\right)$. Moreover, all distances ${\mathrm{HIM}}_{\xi }$ will be nonzero for non-identical isomorphic/isospectral graphs.

### 2.4 A minimal example

**Figure 2:**
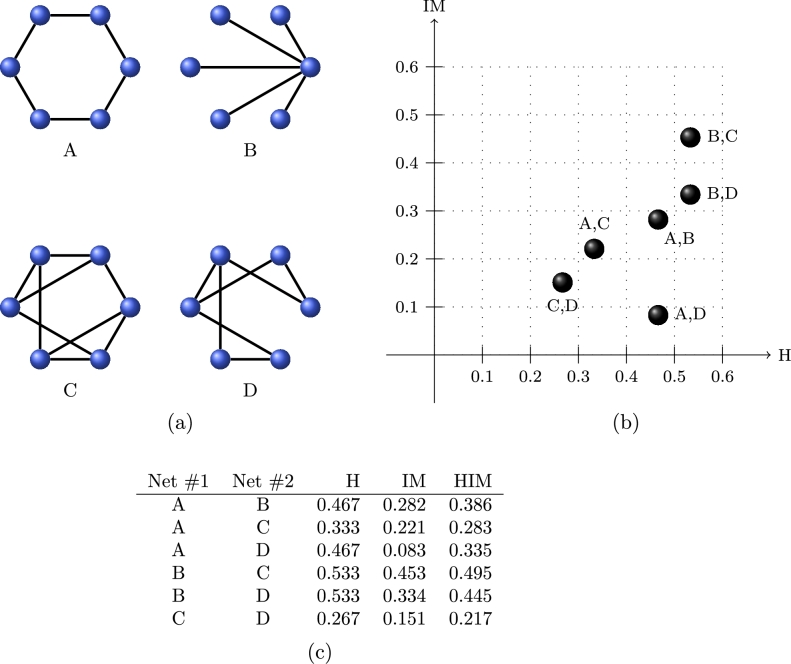
Graphical representation in circular layout (a), scatterplot (b) and tabular (c) representation of the HIM distance in the Ipsen–Mikhailov (IM axis) and Hamming (H axis) distance space between ring network (A), the star network (B), a regular network with degree three (C) and a 3 × 2 regular lattice (D).

In Figure 2 we show, in the H×IM space, the graphical representation in circular layout and the mutual HIM distances between four undirected simple networks on six shared nodes, namely the ring network (A), the star network (B), a regular network with degree three (C) and a 3×2 regular lattice (D). HIM distances range from 0.217 for the pair (C,D), which are the mutually closest networks, to 0.495 for (B,C) which are the farthest graphs. In all cases, the Hamming distance is contributing to the HIM metric more than the Ipsen–Mikhailov component, indicating that the presence or absence of matching links is has a larger impact than the overall structure. Note for instance that networks A and B have the same Hamming distance as A and D, but the spectral structure of the lattice D is closer to the structure of the ring network A than the star network B, as quantitatively shown by the different IM distance; in particular, the spectral structures of A and D are the closest, with IM distance even smaller of the IM distance between C and D. An analogous situation occurs for the pairs B,C and B,D, sharing the same H distance but with a different IM distance.

## 3 Theory

**Figure 3:**
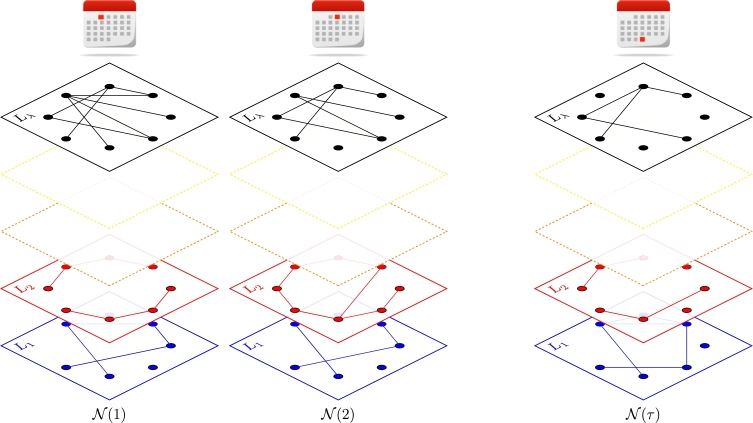
Graphical representation of a sequence N of *τ* multiplex networks N(t) with *λ* layers.

Let $N={\left\{N(t)\right\}}_{t=1}^{\tau }$ be a sequence (time series) of *τ* multiplex networks with *λ* layers ${\left\{L_{i}(t)\right\}}_{i=1}^{\lambda }$ sharing *ν* nodes ${\left\{v_{j}\right\}}_{j=1}^{\nu }$, as displayed in Figure 3.

*The metric projection*. Construct now the metric projection LN(t) of N(t) as the full undirected weighted network with *λ* nodes ${\left\{w_{L_{i}}\right\}}_{i=1}^{\lambda }$ where the weight of the edge connecting vertices $w_{L_{i}}$ and $w_{L_{j}}$ is defined by the HIM similarity between layers $L_{i}(t)$ and $L_{j}(t)$: thus, if $A^{\mathrm{LN}}(t)$ is the adjacency matrix of LN(t), then

$$A_{ij}^{\mathrm{LN}}(t)=1-\mathrm{HIM}(L_{i}(t),L_{j}(t))\;.$$

**Figure 4:**
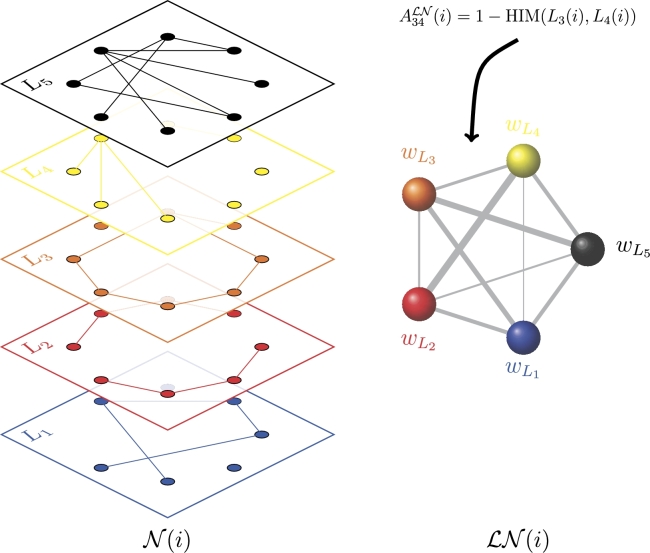
Construction of the metric projection LN(t) at a given time point *t* = *i* for a multiplex network with *λ* = 5 layers; the metric projection is a new network with one node for each layer of the original net, and the edge weight is given by the complement of the HIM distance between the corresponding layers.

In Figure 4 an example of the construction of LN(t) is shown for a multiplex network with five layers.

*The collapsed projection*. Moreover, if $A^{L_{i}}(t)$ is the adjacency matrix of $L_{i}(t)$, define the collapsed projection CN(t) of N(t) on nodes ${\left\{v_{j}\right\}}_{j=1}^{\nu }$ as the network where a link exists between $v_{k}$ and $v_{q}$ if it exists in at least one layer ${\left\{L_{i}(t)\right\}}_{i=1}^{\lambda }$ (for binary layers); in case of weighted layers, the weight of the link $v_{k}-v_{q}$ is the average of the weights across all layers. Thus, if $A^{\mathrm{CN}}(t)$ is the adjacency matrix of CN(t), then

$$A_{kq}^{\mathrm{CN}}(t)=\left\{ \begin{array}{cc} \overset{\lambda }{\underset{i=1}{⋁}}A_{kq}^{L_{i}}(t) & \text{for binary layers}\\ \frac{1}{\lambda }\sum_{i=1}^{\lambda }A_{kq}^{L_{i}}(t) & \text{for weighted layers}\;. \end{array}\right.$$

**Figure 5:**
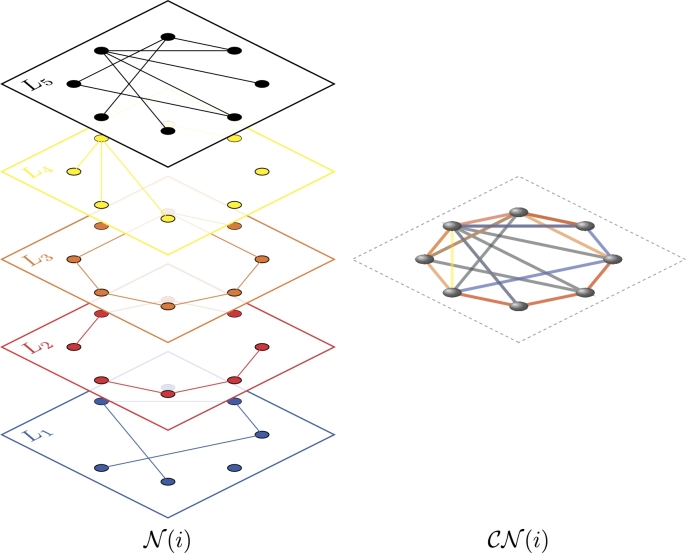
Construction of the collapsed projection CN(t) at a given time point *t* = *i* for a multiplex network with *λ* = 5 layers; the collapsed projection is a new network sharing the same nodes of the original multiplex net, where a link exists in the projection if the same link appears in at least one of the layers of the multiplex network, as if all the layers were collapsed into a single one.

In Figure 5 we show a graphical sketch of the collapsing of a multiplex network with five layers.

Caveat: consider a sequence of binary multiplex networks such that, for each of the possible $\frac{\nu (\nu -1)}{2}$ links and for each timestep, there exists at least one layer including this link. Then the collapsed projection, at each time step, is the full graph on *ν* nodes, and, as such, it has no temporal dynamics, regardless of the evolution of each single layer.

*The distance series*. To investigate the dynamics of N(t) for t=1,…,τ, we construct a suite of associated time series by means of three different procedures, all involving the HIM distance between each network in a given sequence and the first element of the sequence itself. The first group D1 of distance series is obtained by evaluating the dynamics of each layer considered separately:

(D1) $$\left\{\mathrm{HIM}(L_{i}(t),L_{i}(1)),\;t=2,\ldots ,\tau \right\}\;i=1,\ldots ,\lambda \;.$$

**Figure 6:**
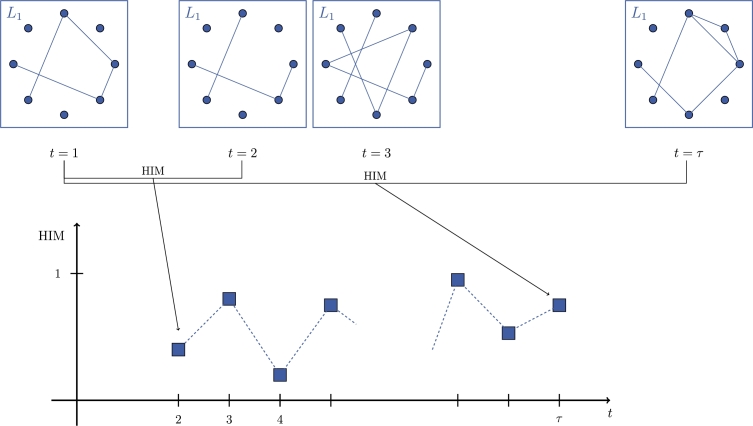
Construction of the distance series D1 for the first layer of the sequence N of multiplex network in Figure 3. The value of the time series at time point *t* = *i* is the HIM distance between the layer *L*_1_ at time *t* = *i* and at time *t* = 1.

In Figure 6 we show the construction of the distance series D1 for the first layer of the multiplex network in Figure 3.

The second series, D2, collects the metric dynamics of the collapsed projection CN:

(D2) HIM(CN(t),CN(1)),t=2,…,τ.

**Figure 7:**
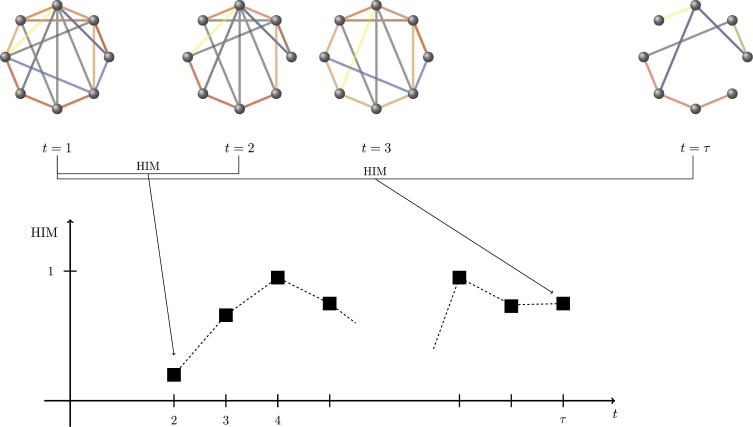
Construction of the distance series D2 for the sequence CN of collapsed networks in Figure 5. The value of the time series at time point *t* = *i* is the HIM distance between CN at time *t* = *i* and at time *t* = 1.

An example of construction of D2 for the five layers multiplex network of Figure 5 is shown in Figure 7.

**Figure 8:**
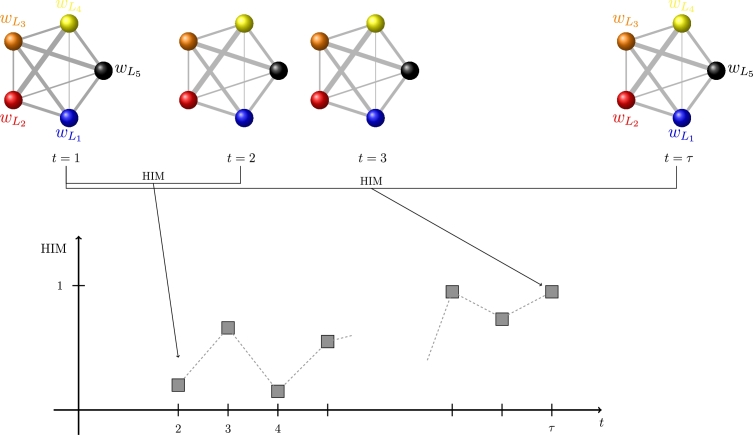
Construction of the distance series D3 for the sequence LN of metric projections in Figure 4. The value of the time series at time point *t* = *i* is the HIM distance between LN at time *t* = *i* and at time *t* = 1.

Finally, the last series D3 collects the metric dynamics of the metric projection LN, and the corresponding example for the multiplex networks in Figure 4 is shown in Figure 8:

(D3) HIM(LN(t),LN(1)),t=2,…,τ.

*Dynamics indicators*. The dynamics of the time series *D*⁎ is quantitatively analyzed by means of a set of indicators, assessing the series' information content and detecting occurring tipping points.

The first indicator is the Increment Entropy (IncEnt), introduced in [21] as a measure the complexity of time series in terms of its unpredictability [30]. The starting point is increment series of a time series as an informative encoder of the characteristics of dynamic changes hidden in a signal. In practice, the increments are grouped in vectors of size *m*, and each increment is mapped into a two-letters word, with a sign and its size coded in this word according to a resolution parameter *R*. Finally, the IncEnt is computed as the Shannon entropy of these words: the larger is the IncEnt value, the less predictable is the series.

The second indicator meanvar belongs to the family of the changepoint detection indicators as implemented in R by the changepoint package [20]. In general, changepoint detection algorithms are the solutions to the problem of estimating the points in a time series where the statistical properties change. The subset of the meanvar functions search for changes in both the mean and the variance, and a number of alternative are known in literature [31], [32], [33], [34], [35], [36]. In particular, in what follows we will employ the recent Pruned Exact Linear Time (PELT) algorithm [37], based on the classical segment neighborhood technique minimizing the combination of a cost function (for instance, twice the negative log-likelihood) with a linear penalty function through dynamic programming. Finally, we will use Changepoints for a Range of PenaltieS (CROPS) [38] to obtain optimal changepoint segmentations of data sequences for all penalty values across a continuous range.

## 4 Results & discussion

### 4.1 A synthetic example

Consider now a sequence of binary multiplex networks with τ=30, λ=5 and ν=10, generated as follows.

Define the perturbation function Π(N,(m,M)) taking as entries a binary simple network *N* on *n* nodes, and a couple of real values (m,M) with 0≤m≤M≤1, and returning a network $N^{\prime }$ obtained from *N* by swapping the status (present/not present) of $\left\lfloor g\frac{n(n-1)}{2}\right\rfloor$ links, where *g* is a random value in the interval [m,M]. Further, define the default transition as the pair $\sigma_{d}=(0.05,0.2)$, a small transition as $\sigma_{s}=(0.2,0.3)$, a medium transition as $\sigma_{m}=(0.25,0.4)$ and, finally, a large transition as $\sigma_{l}=(0.5,0.7)$. Moreover, let *R* be an Erdós–Rényi G(ν,0.3) random model and define 4 special timepoints: the initial time step $\tau_{0}=1$, the first spike $\tau_{1}=10$, the second spike $\tau_{2}=17$ and the third spike $\tau_{3}=24$.

Then, each layer $L_{i}$ at a given time step *t* is defined through the following rule:

$$L^{i}(t)=\left\{ \begin{array}{cc} \Pi (R,\sigma_{s}) & \text{if}\;t=\tau_{0}\;\text{and}\;i=1,2\\ \Pi (R,\sigma_{m}) & \text{if}\;t=\tau_{0}\;\text{and}\;i=3,4\\ \Pi (R,\sigma_{l}) & \text{if}\;t=\tau_{0}\;\text{and}\;i=5\\ \Pi (L^{i}(t-1),\sigma_{s}) & \text{if}\;t=\tau_{1}\;\text{and}\;i=1,3,5\\ & \text{or if}\;t=\tau_{2}\;\text{and}\;i=3,5\\ & \text{or if}\;t=\tau_{3}\;\text{and}\;i=5\\ \Pi (L^{i}(t-1),\sigma_{m}) & \text{if}\;t=\tau_{2}\;\text{and}\;i=1,2\\ & \text{or if}\;t=\tau_{3}\;\text{and}\;i=3\\ \Pi (L^{i}(t-1),\sigma_{l}) & \text{if}\;t=\tau_{1}\;\text{and}\;i=2,4\\ & \text{or if}\;t=\tau_{2}\;\text{and}\;i=4\\ & \text{or if}\;t=\tau_{3}\;\text{and}\;i=1,2,4\\ \Pi (L^{i}(t-1),\sigma_{d}) & \text{otherwise}\;. \end{array}\right.$$

**Figure 9:**
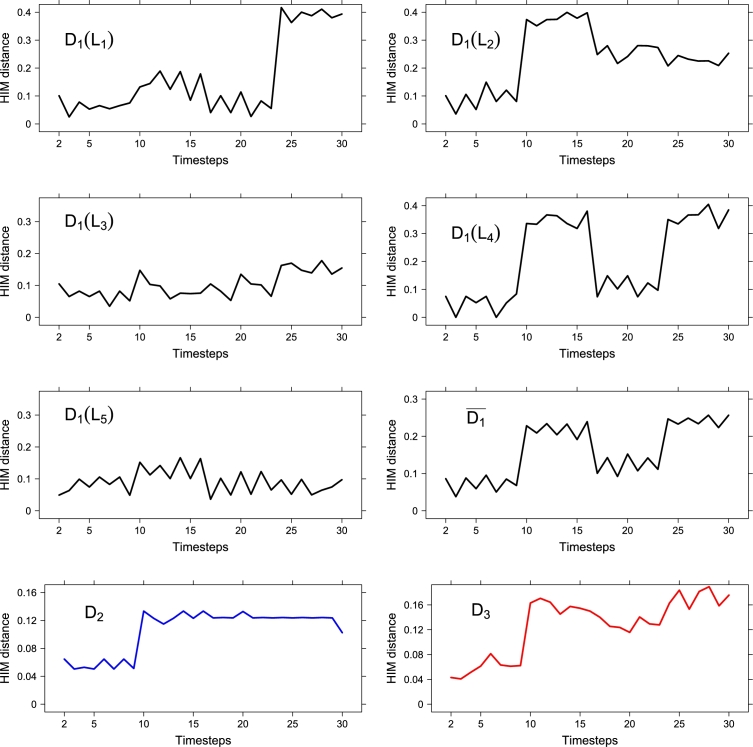
*D*_1_, *D*_2_, *D*_3_ for a synthetic example on 5 layers and 30 timepoints; in the right column, third row, we plot ${\bar{D}}_{1}=\frac{1}{5}\sum_{i=1}^{5}D_{i}(L_{i})$.

In Figure 9 we show the evolution along the 30 timepoints of the 5 curves for $D_{1}(L_{i})$, its average ${\bar{D}}_{1}=\frac{1}{5}\sum_{i=1}^{5}D_{i}(L_{i})$ and $D_{2}$, $D_{3}$. To assess the information content of each curve we use the Increment Entropy indicator IncEnt, whose value increases with the series' complexity: the IncEnt values are reported in Table 1.

Among the evolving layers, $L_{2}$ and $L_{4}$ have the largest IncEnt, while the other three layers show a lower level of complexity. As expected, the average $\bar{D_{1}}$ and the collapsed network distance $D_{2}$ has very low IncEnt value, yielding that both averaging the distances and collapsing the layers lose information about the overall dynamics. Finally, distance $D_{3}$ is the metric which better detects the network evolution along time, conserving most of the information. This is also supported by the meanvar indicator with CROPS range [2log⁡(τ),10log⁡(τ)] with the PELT algorithm: in fact, the meanvar indicator detects correctly in $D_{3}$ the three points $\tau_{1}-1$, $\tau_{2}-1$ and $\tau_{3}-1$ as changepoints, while in $D_{2}$, other than the $\tau_{1}-1$, meanvar detects t=20 and =28 which are unrelated to the designed dynamics.

### 4.2 The Gulf Dataset

*Data description*.

Part of the Penn State Event Data http://eventdata.psu.edu/ (formerly Kansas Event Data System), available at http://vlado.fmf.uni-lj.si/pub/networks/data/KEDS/, the Gulf Dataset collects, on a monthly bases, political events between pairs of countries focusing on the Gulf region and the Arabian peninsula for the period 15 April 1979 to 31 March 1999, for a total of 240 months. The 304401 political events involve 202 countries and they belong to 66 classes (including for instance “pessimist comment”, “meet”, “formal protest”, “military engagement”, etc.) as coded by the World Event/Interaction Survey (WEIS) Project [39], [40], [41] http://www.icpsr.umich.edu/icpsrweb/ICPSR/studies/5211, whose full list is reported in Table 2, 3.

In the notation of Sec. 3, the Gulf Dataset translates into a time series of τ=240 multiplex networks with λ=66 unweighted and undirected layers sharing ν=202 nodes. The landmark event for the considered zone in the 20 years data range of interest is definitely the First Gulf War (FGW), occurring between August 1990 and March 1999. However, other (smaller) events located in the area had a relevant impact on world politics and diplomatic relations. Among them, the Iraq Disarmament Crisis (IDC) in February 1998 significantly emerges from the data, as shown in what follows. During that month, Iraq President Saddam Hussein negotiated a deal with U.N. Secretary General Kofi Annan, allowing weapons inspectors to return to Baghdad, preventing military action by the United States and Britain.

### 4.3 Network statistics

Consider in this section the set of 304401 edges connecting the 202 nodes independently of their class. In Table 4 we list the top-10 countries/institutions participating in the largest number of edges across different time spans, together with the absolute number of shared edges and the corresponding percentage over the total number of edges for the period. In general, USA, Iraq and Iran are the major players, with different proportions according to the specific period: in particular, Iraq is the main character in both the major events, FGW and IDC. Other key actors are Israel, the United Nations and the Saudi Arabia, with a relevant presence in each key event in the area. Note that, overall, the top 20 institutions (also including, other than those listed in the table, the Arab world, France, Syria, Egypt, Russia, Turkey, Jordan, Libya, Germany and the Kurd world) are responsible for 82.57% of all edges.

Out of all potential $\frac{202\cdot 201}{2}=20301$ unique edges, only 4394 are represented in the Gulf Dataset. In Table 5 we list the top-10 links ranked by occurrence, together with the number of occurrences itself and the corresponding percentage over the total number of edges for the period. As it happens for the nodes, there are a few key links throughout the whole timespan which are consistently present in most of the important events, with different proportions. However, in some of the events, there is an interesting wide gap in the number of occurrences between the very top edges and the remaining ones, *e.g.*, Iraq–USA in FGW (and post) and IDC, and Iran–Iraq during the corresponding war and in the pre-FGW, yielding that these are the links mainly driving the whole network evolution.

**Figure 10:**
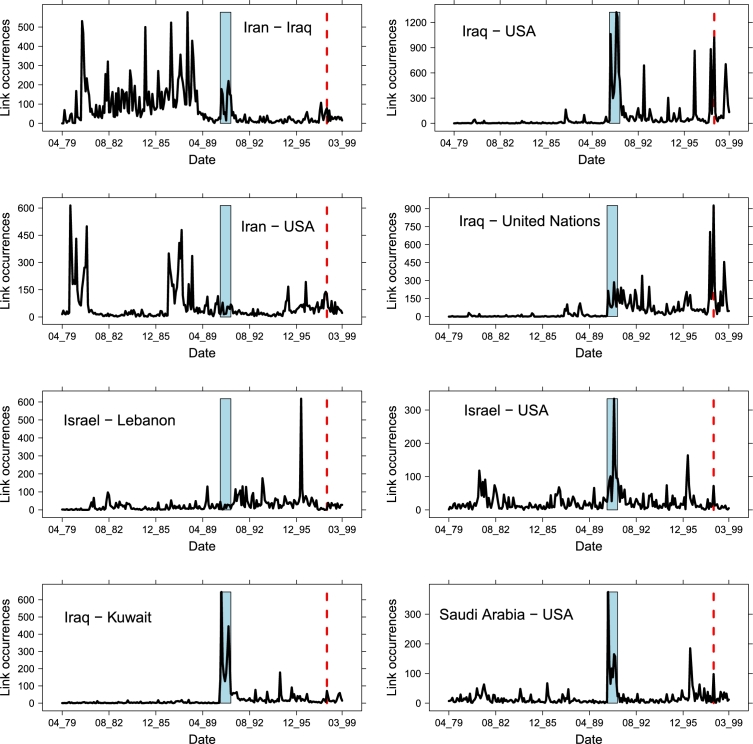
Occurrences along time of the top-8 most frequent links. The blue area marks FGW, while the red dashed line indicates IDC in February 98.

In Figure 10 we display the dynamics of the occurrence along time of the top edges, showing their different trends during the diverse events. It is interesting to note how two top links, Iran–Iraq and Iran–USA are preponderant from 1979 to 1989, *i.e.*, throughout the whole Iran–Iraq War, while they go decaying quickly afterwards, with a minor spike for FGW. Complementarily, two other major links Iraq–USA and Iraq–United Nations have the opposite trend, remaining almost uninfluential until FGW and growing later on, with a noticeable spike for IDC; moreover, Iraq–United Nations does not show any trend change for FGW, while Iraq–USA does. The Iraq–Kuwait link has a very limited dynamics, with the unique important spike for FGW. Very similar are also the Saudi Arabia–USA and the Israel–USA links, showing an additional lower spike in correspondence of the raise of the terroristic actions between 1995–1996. This last event is crucial in the Israel–Lebanon relations, where it has the largest effect; FGW, instead, has almost no impact here.

**Figure 11:**
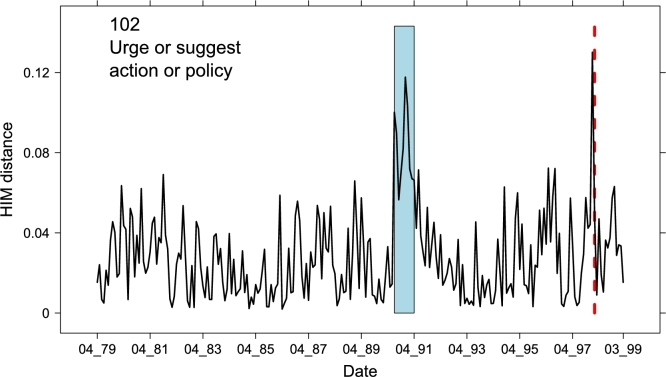
*D*_1_ time series for the layer 37, corresponding to WEIS code 102 (“Urge or suggest action or policy”). The period corresponding to FGW is marked by the blue background, while the red dashed line indicates IDC in February 1998.

**Figure 12:**
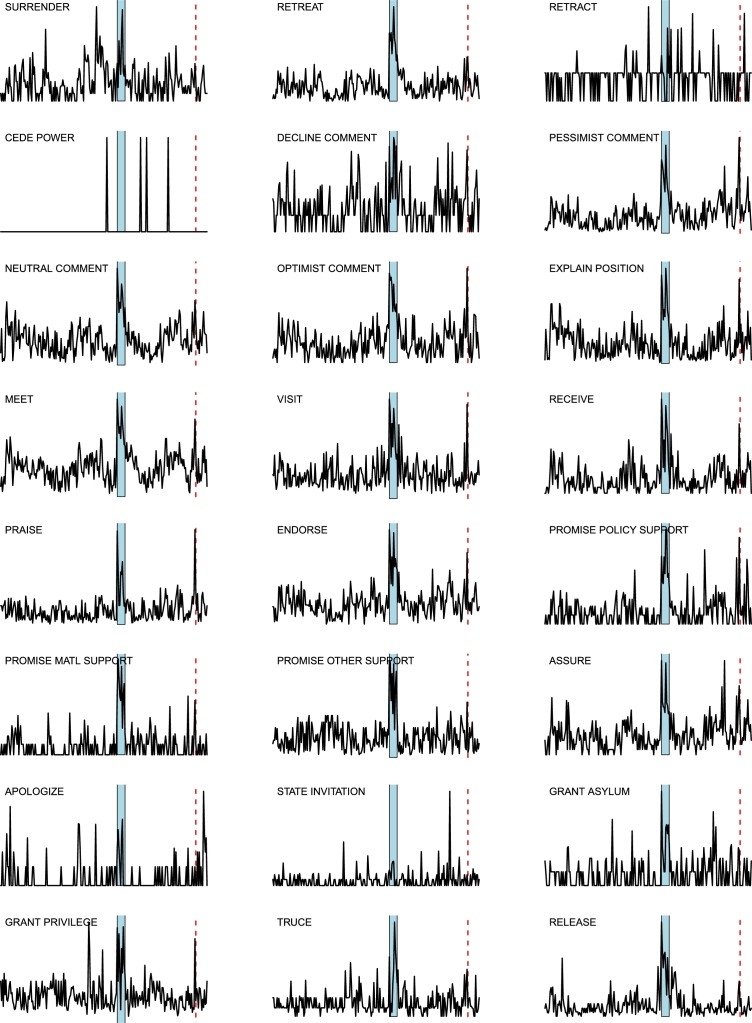
Curves of indicator *D*1 for the 24 layers *L*_*i*_(*t*), for *i* = 1,…,24: the blue area marks FGW, while the red dashed line indicates IDC in February 98. For each curve, the corresponding World Event/Interaction Survey category is indicated in the top left corner.

**Figure 13:**
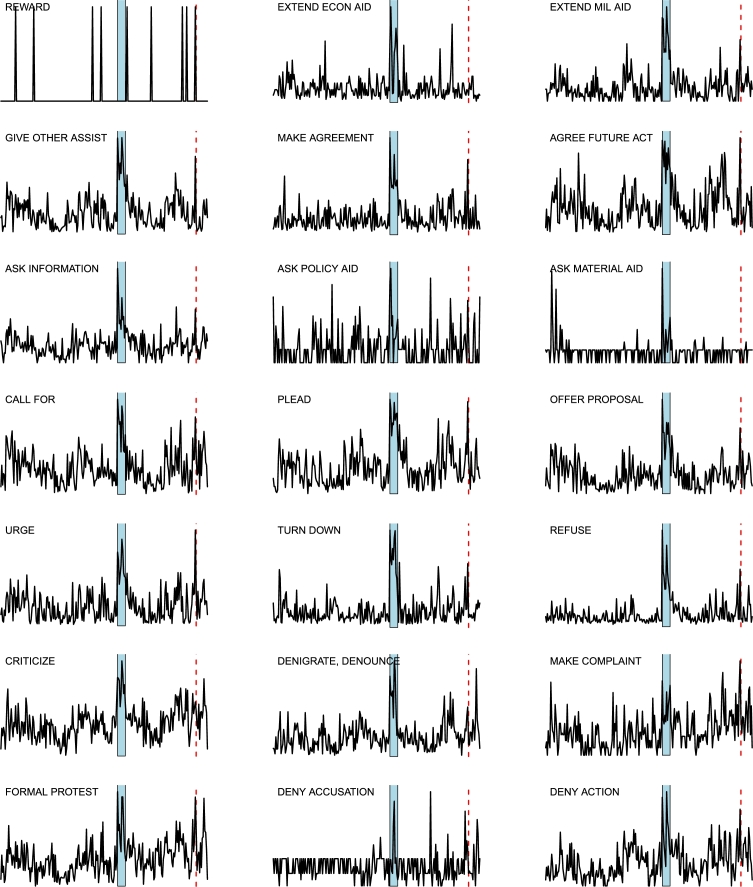
Curves of indicator *D*1 for the 21 layers *L*_*i*_(*t*), for *i* = 25,…,45: the blue area marks FGW, while the red dashed line indicates IDC in February 98. For each curve, the corresponding World Event/Interaction Survey category is indicated in the top left corner.

*D*⁎ *indicators analysis*. The two main events FGW and IDC generate sudden changes in the D1 time series for most of the layers: an example is given in Figure 11 for the layer 37, corresponding to WEIS code 102 (“Urge or suggest action or policy”), where we highlight FGW by a blue background, and IDC by a red dashed line. The complete panel of the $D_{1}$ curves for all the 66 layers is shown in Figure 12,13,14: most of the layers show a decise change in trend in correspondence of the two main events, although some of the layers display a different behavior (*e.g.*, layer 59, “Break diplomatic relations”), sometimes due to the paucity of data (*e.g.*, “Halt negotiations” or “Reward”). Note that many other spikes exist in many layers, corresponding to different geopolitical events occurring throughout the considered timespan.

All the information conveyed by the 66 $D_{1}$ time series can be summarized by using the $D_{2}$ and $D_{3}$ indicators displayed in Figure 15. The two curves show a similar trend, with two major spikes corresponding to the FGW and the IDC, neatly emerging in both time series. Furthermore, both indicators are consistent in showing that the two periods pre- and post-FGW are not part of the FGW spike, implying that in these two periods the structure of the occurring binary geopolitical events is closer to the analogous structure for the “no-war” periods.

**Figure 14:**
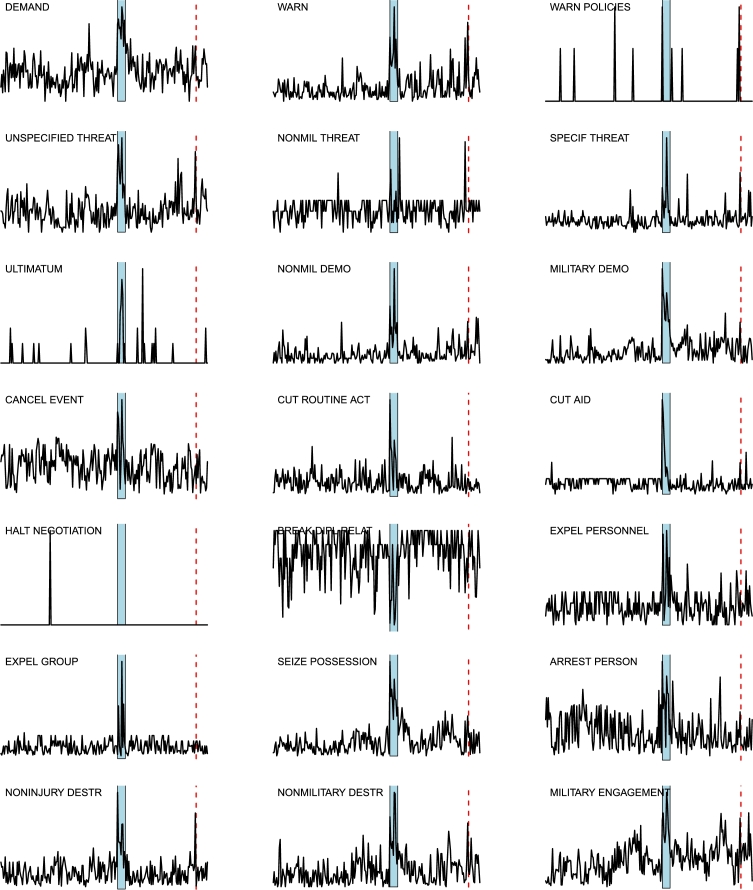
Curves of indicator *D*1 for the 21 layers *L*_*i*_(*t*), for *i* = 46,…,66: the blue area marks FGW, while the red dashed line indicates IDC in February 98. For each curve, the corresponding World Event/Interaction Survey category is indicated in the top left corner.

**Figure 15:**
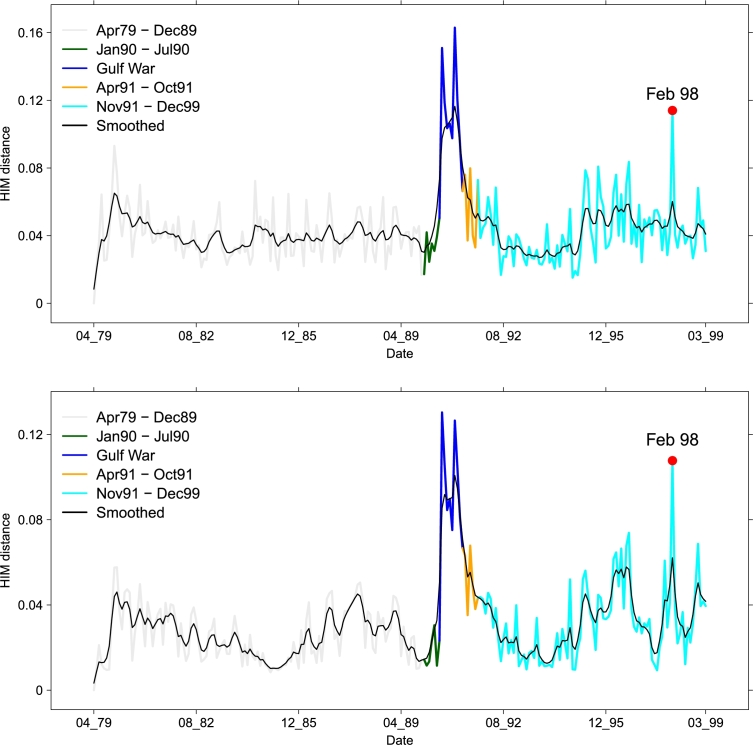
Time evolution of a global view of the (monthly) Gulf Dataset. (top) *D*_2_ dynamics of the collapsed projections ${\left\{\mathrm{CN}(t)\right\}}_{t=1}^{240}$ and (bottom) *D*_3_ dynamics of the metric projections ${\left\{\mathrm{LN}(t)\right\}}_{t=1}^{240}$. For each date, the value on y-axis is the HIM distance from the first element of the time series. Different colors mark different time periods. The black line represents the fixed-interval smoothing via a state-space model [48].

However, as expected, the indicator $D_{2}$ includes a lower level of information than $D_{3}$: this is particularly evident (also for the smoothed curves, in black in the plots) in the periods 85–89 and 95–97, where the dynamics of $D_{2}$ is much flatter than the dynamics of $D_{3}$. Note that a nontrivial dynamics in the two periods 85–89 and 95–97 exist in many layers, as shown in Figure 12,13,14, triggered by a number of important events impacting the geopolitical relations: the final part of the Iran–Iraq War (1980–1988), the decline and fall of the Soviet Empire (not directly related to the Middle East area, but reflecting also there), the dramatic change of the situation of the Middle East conflicts induced by the outbreak of the First Intifada in December 1987 [42], and the terrorism excalation (Dhahran, Tel Aviv, and Jerusalem) in Middle East in 95/96 causing a bursting increase in the number of victims just to name the more relevant events.

Thus, this case study, too supports the superiority of $D_{3}$ as a global indicator to summarize the evolution of a series of multiplex networks.

**Figure 16:**
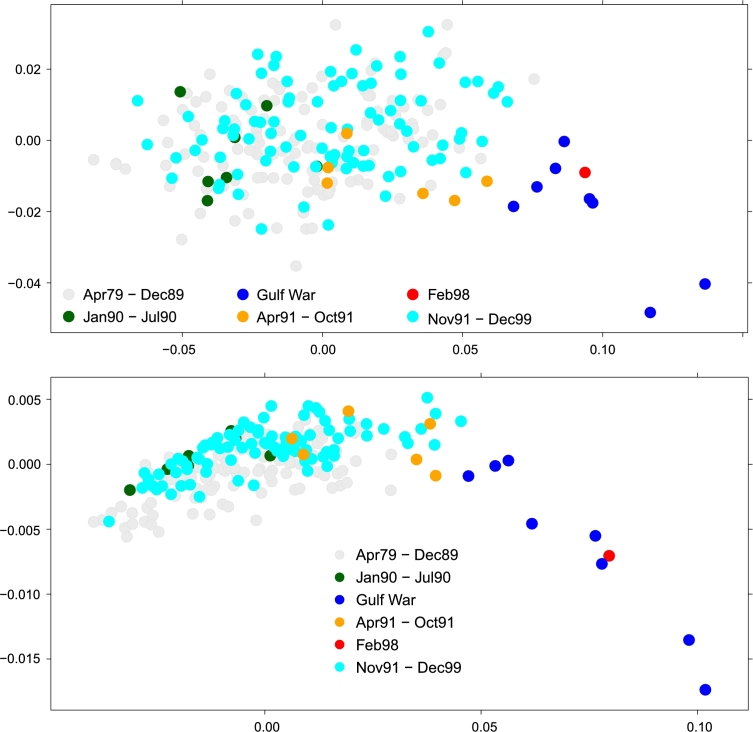
Planar multidimensional scaling plot with HIM distance of the collapsed (top) and metric (bottom) projection for the monthly Gulf Dataset. Colors are consistent with those in Figure 15.

We also computed all the $\frac{240\cdot 239}{2}$ HIM distances for $D_{2}$ (respectively, $D_{3}$) ${\left\{\mathrm{HIM}(\mathrm{CN}(t_{i}),\mathrm{CN}(t_{j}))\right\}}_{1\leq i\leq j\leq \tau =240}$ (resp. ${\left\{\mathrm{HIM}(\mathrm{LN}(t_{i}),\mathrm{LN}(t_{j}))\right\}}_{1\leq i\leq j\leq \tau =240}$), which are then used to project the 240 networks on a plane through a MultiDimensional Scaling (MDS) [43]: the resulting plots are displayed in Figure 16.

Both indicators yield that the months corresponding to FGW (in blue in the plots) are close together and confined in the lower left corner of the plane, showing both a mutual high degree of homogeneity and, at the same time, a relevant difference to the graphs of all other months. Interestingly, this holds also for the months immediately before and after (in green and orange in the figures) the conflict, which are quite distant from the war months' cloud, as previously observed. This confirms that, only at the onset of the conflict the diplomatic relations worldwide changed consistently and their structure remained very similar throughout the whole event.

From both the multidimensional scaling plots in Figure 16 it is clear that the both the CN and LN networks for the FGW months can be easily discriminated from all other nets. However, from the MDS projections it is not evident whether the months Apr 1979 – Dec 1989 (in grey) could be separated from the Nov 91 – Dec 99 months. By using a Support Vector Machine classifier with the HIM kernel [10], [25] (with γ=172.9 for LN and γ=110 for CN), a 5-fold CV classification gives as best result the accuracy 81.2% for LN ($C=10^{3}$) and 73.3% for CN ($C=10^{4}$). Thus, in both cases, machine learning provides a good separation between the networks belonging to the two periods.

**Figure 17:**
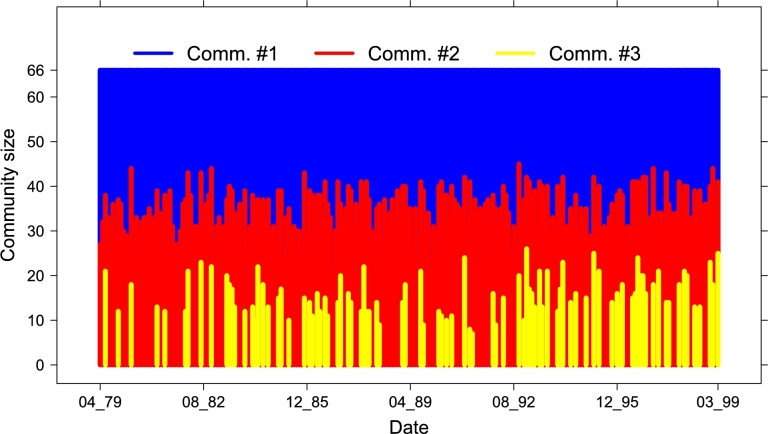
Dimension of the three communities identified by the Louvain algorithm in LN along the 240 months.

**Figure 18:**
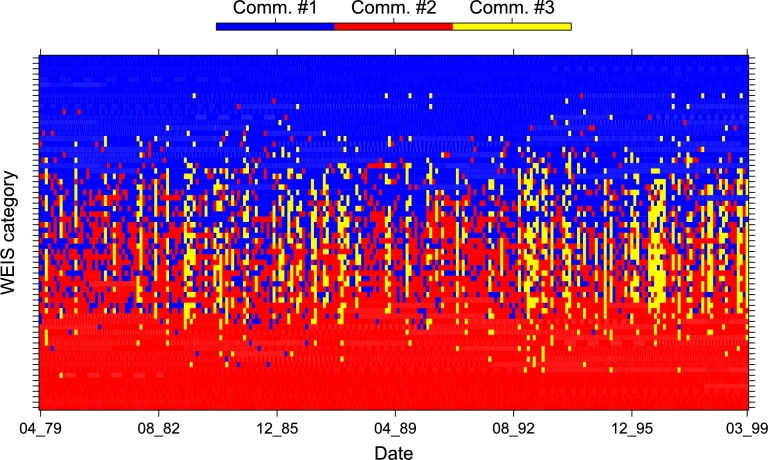
Community evolution along time for each of the 66 WEIS categories, ranked by community distribution.

**Figure 19:**
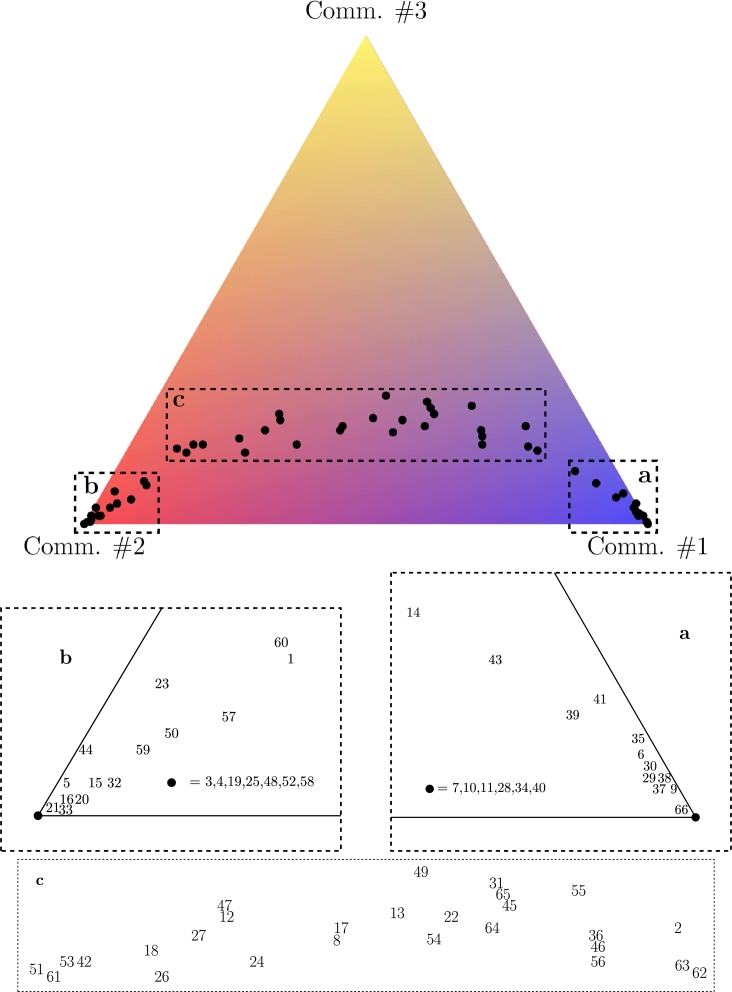
Triangleplot projection of the 66 WEIS categories defined by their community distribution.

*Community structure of* LN. We conclude by analyzing the dynamics of the mesostructure of the layer network LN as extracted by the Louvain community detection algorithm [44], [45], [46], [47]. For any temporal step, the Louvain algorithm clusters the 66 nodes (WEIS categories) of LN into two or three communities, whose dimension along time is shown in Figure 17. In Figure 18 we show, for each date, which community each category (on the rows) belongs to; WEIS categories are ranked according to their community distribution, *i.e.*, decreasing number of presences in Comm. #1 and increasing for Comm. #2. Thus in top rows we have the categories lying in Comm. #1 during all the 240 months (layers 7,10,11,28,34,40), while bottom rows are reserved to the categories always belonging to Comm. #2 (3,4,19,25,48,52,58): their description in terms of WEIS categories is shown in Table 6, while the full community distribution is reported in Table 7 and graphically summarized by the triangleplot in Figure 19. Focusing on the categories that are consistently lying in a given community throughout all 240 months, some of them are semantically similar: for instance, consult, assistance, action request in community #1 while two distinct groups emerge in community #2, namely admit wrongdoing, cede power, apologize, reward on one side and warn of policies, sanction threats and halt negotiations characterizing the second group. However, it is interesting the constant presence of the category charge/criticize/blame/disapprove in community #1. Moreover, there is no strong polarization for Community #3. Many layers sharing the same (or similar) WEIS second level category (Yield, Comment, Consult, etc.) are quite close in the community distribution ranked list, with a general escalating trend proceedings from help request (or other more neutral actions) to more severe situations growing together with the community distribution rank.

## 5 Conclusion

We introduced here a novel approach for the longitudinal analysis of a time series of multiplex networks, defined by mean of a metric transformation conveying the information carried by all layers into a single network for each timestamp, with the original layers as nodes. The transformation is induced by the Hamming–Ipsen–Mikhailov distance between graph sharing the same nodes, and it preserves the key events encoded into each instance of the multiplex network time series, making it more efficient than the collapsing of all layers into one collecting all edges for detecting important fluctuations in the original network's dynamics. Moreover, a community detection analysis on the obtained network can help shading light on the relations between the original layers throughout the whole time span.

## Declarations

### Author contribution statement

Giuseppe Jurman: Conceived and designed the experiments; Performed the experiments; Analyzed and interpreted the data; Contributed reagents, materials, analysis tools or data; Wrote the paper.

### Funding statement

This research did not receive any specific grant from funding agencies in the public, commercial, or not-for-profit sectors.

### Competing interest statement

The authors declare no conflict of interest.

### Additional information

Data associated with this study has been deposited at http://vlado.fmf.uni-lj.si/pub/networks/data/KEDS/GulfLDays.zip.

## Figures and Tables

**Table 1:** Increment Entropy values for the distance sequences of the synthetic example, with parameters *m* = 2, *R* = 2.

Dist.	IncEnt	Dist.	IncEnt
*D*_1_(*L*_1_)	2.52	*D*_1_(*L*_2_)	2.78
*D*_1_(*L*_3_)	2.59	*D*_1_(*L*_4_)	2.83
*D*_1_(*L*_5_)	2.44	${\bar{D}}_{1}$	2.27
*D*_2_	1.82	*D*_3_	3.04

**Table 2:** Part 1 of the full table of WEIS codes [39], with the 66 layers considered in the Gulf dataset case study; entries with no layer number were not monitored in the Gulf dataset events collection.

Layer#	WEIS code	WEIS cat	Description
1	011	Yield	Surrender, yield or order, submit to arrest, etc.
2	012	Yield	Yield position, retreat; evacuate
3	013	Yield	Admit wrongdoing; retract statement
	014	Yield	Accommodate, Cease-fire
4	015	Yield	Cede Power
5	021	Comment	Explicit decline to comment
6	022	Comment	Comment on situation – pessimistic
7	023	Comment	Comment on situation – neutral
8	024	Comment	Comment on situation – optimistic
9	025	Comment	Explain policy or future position
	026	Comment	Appoint or Elect
	027	Comment	Alter Rules
10	031	Consult	Meet with at neutral site, or send note.
11	032	Consult	Consult & Visit; go to
12	033	Consult	Receive visit; host
	034	Consult	Vote, Elect
13	041	Approve	Praise, hail, applaud, condole
14	042	Approve	Endorse other's policy or position; give verbal support
	043	Approve	Rally
15	051	Promise	Promise own policy support
16	052	Promise	Promise material support
17	053	Promise	Promise other future support action
18	054	Promise	Assure; reassure
	055	Promise	Promise Rights
19	061	Grant	Express regret; apologize
20	062	Grant	Give state invitation
21	063	Grant	Grant asylum
22	064	Grant	Grant privilege, diplomatic recognition
23	065	Grant	Suspend negative sanctions; truce
24	066	Grant	Release and/or return persons or property
	067	Grant	Grant Position
25	070	Reward	Reward
26	071	Reward	Extend economic aid (as gift and/or loan)
27	072	Reward	Extend military assistance
28	073	Reward	Give other assistance
29	081	Agree	Make substantive agreement
30	082	Agree	Agree to future action or procedure; agree to meet, to negotiate
	083	Agree	Ally
	084	Agree	Merge; Integrate
31	091	Request	Ask for information
32	092	Request	Ask for policy assistance
33	093	Request	Ask for material assistance
34	094	Request	Request action; call for
35	095	Request	Entreat; plead; appeal to
	096	Request	Request policy change
	097	Request	Request rights

**Table 3:** Part 2 of the full table of WEIS codes [39], with the 66 layers considered in the Gulf dataset case study; entries with no layer number were not monitored in the Gulf dataset events collection.

Layer#	WEIS code	WEIS cat	Description
36	101	Propose	Offer proposal
37	102	Propose	Urge or suggest action or policy
38	111	Reject	Turn down proposal; reject protest demand, threat, etc
39	112	Reject	Refuse; oppose; refuse to allow
	113	Reject	Defy law
40	121	Accuse	Charge; criticize; blame; disapprove
41	122	Accuse	Denounce; denigrate; abuse
	123	Accuse	Investigate
42	131	Protest	Make complaint (not formal)
43	132	Protest	Make formal complaint or protest
	133	Protest	Symbolic act
44	141	Deny	Deny an accusation
45	142	Deny	Deny an attributed policy, action role or position
46	150	Demand	Issue order or command; insist; demand compliance; etc
	151	Demand	Issue Command
	152	Demand	Claim Rights
47	160	Warn	Give warning
48	161	Warn	Warn of policies
	162	Warn	Warn of problem
49	171	Threaten	Threat without specific negative sanctions
50	172	Threaten	Threat with specific non-military negative sanctions
51	173	Threaten	Threat with force specified
52	174	Threaten	Ultimatum; threat with negative sanctions and time limit specified
53	181	Demonstrate	Non-military demonstration; to walk out on
54	182	Demonstrate	Armed force mobilization
55	191	Reduce Relations^1^	Cancel or postpone planned event
56	192	Reduce Relations^1^	Reduce routine international activity; recall officials; etc
57	193	Reduce Relations^1^	Reduce or halt aid
58	194	Reduce Relations^1^	Halt negotiations
59	195	Reduce Relations^1^	Break diplomatic relations
	196	Reduce Relations^1^	Strike
	197	Reduce Relations^1^	Censor
60	201	Expel	Order personnel out of country
61	202	Expel	Expel organization or group
	203	Expel	Ban Organization
62	211	Seize	Seize position or possessions
63	212	Seize	Detain or arrest person(s)
	213	Seize	Hijack; Kidnap
64	221	Force	Non-injury obstructive act
65	222	Force	Non-military injury-destruction
66	223	Force	Military engagement

1 As negative sanctions.

**Table 4:** Top-10 countries/institutions ranked by number of shared links, absolute and in percentage over (twice) the total number of links in the considered period. The Iran–Iraq War started in September 1980 and ended in August 1988. SA: Saudi Arabia; UN: United Nations.

Apr79–Mar99			FGW			IDC		
Edges 304401			Edges 41181			Edges 7712		
Inst.	Degree	%	Inst.	Degree	%	Inst.	Degree	%
USA	93900	15.42	Iraq	18691	22.69	Iraq	3830	24.83
Iraq	84974	13.96	USA	15584	18.92	USA	2876	18.65
Iran	61782	10.15	Kuwait	5245	6.37	UN	1946	12.62
Israel	32204	5.29	SA	3548	4.31	Russia	896	5.81
UN	30097	4.94	Israel	3420	4.15	UK	715	4.64
SA	20503	3.37	UN	3363	4.08	France	651	4.22
Lebanon	19130	3.14	UK	2997	3.64	Iran	468	3.03
Palestine	18607	3.06	Iran	2104	2.55	Arab world	321	2.08
UK	18415	3.02	France	2076	2.52	China	309	2.00
Kuwait	17405	2.86	Arab world	2053	2.49	Kuwait	306	1.98



Apr79-FGW			FGW-Mar99			Iran–Iraq War		
Edges 130990			Edges 132230			Edges 95189		
Inst.	Degree	%	Inst.	Degree	%	Inst.	Degree	%
Iran	43818	16.73	USA	43606	16.49	Iran	32812	17.24
USA	34710	13.25	Iraq	40677	15.38	USA	24111	12.66
Iraq	25606	9.77	UN	19858	7.51	Iraq	21019	11.04
Israel	12731	4.86	Israel	16053	6.07	Israel	9189	4.83
Palestine	10622	4.05	Iran	15860	6.00	SA	8089	4.25
Lebanon	10374	3.96	UK	9209	3.48	Palestine	7521	3.95
SA	10290	3.93	Lebanon	8143	3.08	Lebanon	6992	3.67
Arab world	8237	3.14	France	6925	2.62	Syria	6072	3.19
Syria	8089	3.09	Russia	6875	2.60	Arab world	5726	3.01
UN	6876	2.62	SA	6665	2.52	Kuwait	4890	2.57

**Table 5:** Top-10 countries/institutions ranked by number of shared links, absolute and in percentage over (twice) the total number of links in the considered period. The Iran–Iraq War started in September 1980 and ended in August 1988. SA: Saudi Arabia; UN: United Nations.

Apr79–Mar99			FGW			IDC		
Edges 304401			Edges 41181			Edges 7712		
Edge	Degree	%	Edge	Degree	%	Edge	Degree	%
Iran–Iraq	19121	6.28	Iraq–USA	6061	14.72	Iraq–USA	1021	13.24
Iraq–USA	19002	6.24	Iraq–Kuwait	2306	5.60	Iraq–UN	927	12.02
Iran–USA	14051	4.62	SA–USA	1169	2.84	UN–USA	337	4.37
Iraq–UN	12775	4.20	Iraq–UN	1118	2.71	Iraq–Russia	315	4.08
Israel–Lebanon	6590	2.16	Kuwait–USA	1050	2.55	Iraq–UK	241	3.12
Israel–USA	5803	1.91	Iraq–UK	1012	2.46	France–Iraq	191	2.48
Iraq–Kuwait	5187	1.70	Iran–Iraq	989	2.40	UK–USA	184	2.39
SA–USA	4468	1.47	Israel–USA	935	2.27	France–UN	171	2.22
Israel–Palestina	4466	1.47	Iraq–Israel	851	2.07	Russia–USA	170	2.20
UN–USA	4209	1.38	Iraq–SA	796	1.93	Iraq–Turkey	136	1.76



Apr79-FGW			FGW-Mar99			Iran–Iraq War		
Edges 130990			Edges 132230			Edges 95189		
Edge	Degree	%	Edge	Degree	%	Edge	Degree	%
Iran–Iraq	16015	12.23	Iraq–USA	11647	8.81	Iran–Iraq	14470	15.20
Iran–USA	9928	7.58	Iraq–UN	10605	8.02	Iran–USA	6456	6.78
Israel–USA	2714	2.07	Israel–Lebanon	4471	3.38	Israel–USA	2124	2.23
Iran–UN	2105	1.61	Iran–USA	3797	2.87	Iran–UN	1402	1.47
Israel–Lebanon	1981	1.51	UN–USA	2575	1.95	Israel–Lebanon	1391	1.46
Israel–Palestina	1932	1.47	Iraq–Kuwait	2371	1.79	Lebanon–USA	1327	1.39
Lebanon–USA	1722	1.31	UK-U-SA	2206	1.67	SA–USA	1271	1.34
Lebanon–Syria	1591	1.21	Israel–USA	2154	1.63	Israel–Palestina	1247	1.31
SA–USA	1554	1.19	Israel–Palestina	2144	1.62	France–Iran	1193	1.25
France–Iran	1418	1.08	Iran–Iraq	2117	1.60	Lebanon–Syria	999	1.05

**Table 6:** The 13 layers not swapping community across all 240 timepoints.

Community #1		
Layer	WEIS code	WEIS category
7	023	[Comment] Comment on situation – neutral
10	031	[Consult] Meet with at neutral site, or send note
11	032	[Consult] Consult & Visit; go to
28	073	[Reward] Give other assistance
34	094	[Request] Request action; call for
40	121	[Accuse] Charge; criticize; blame; disapprove


Community #2		
Layer	WEIS code	WEIS category
3	013	[Yield] Admit wrongdoing; retract statement
4	015	[Yield] Cede power
19	061	[Grant] Express regret; apologize
25	070	[Reward] Reward
48	161	[Warn] Warn of policies
52	174	[Threaten] Ultimatum; threat with negative sanctions and time limit specified
58	194	[Reduce Relations] Halt negotiations

**Table 7:** Community distribution for the 66 WEIS categories: for each layer, we report the number of occurrences in the three detected communities.

Rank	Layer	#1	#2	#3	Rank	Layer	#1	#2	#3
1	7	240	0	0	34	8	86	108	46
2	10	240	0	0	35	17	86	106	48
3	11	240	0	0	36	24	71	130	39
4	28	240	0	0	37	12	58	131	51
5	34	240	0	0	38	47	56	130	54
6	40	240	0	0	39	27	54	140	46
7	66	239	0	1	40	26	51	154	35
8	9	236	0	4	41	18	45	153	42
9	37	234	2	4	42	42	31	170	39
10	38	234	1	5	43	53	27	174	39
11	29	233	2	5	44	61	26	179	35
12	30	232	2	6	45	51	21	182	37
13	6	230	2	8	46	1	17	204	19
14	35	230	0	10	47	60	15	204	21
15	41	222	3	15	48	57	14	214	12
16	39	220	7	13	49	50	9	221	10
17	43	208	12	20	50	59	7	225	8
18	14	196	18	26	51	23	5	219	16
19	62	175	29	36	52	32	5	231	4
20	63	170	32	38	53	15	4	232	4
21	2	164	28	48	54	16	2	236	2
22	56	150	51	39	55	20	2	236	2
23	46	148	49	43	56	33	2	237	1
24	36	146	48	46	57	5	1	235	4
25	55	136	46	58	58	21	1	238	1
26	45	122	64	54	59	44	1	231	8
27	64	121	71	48	60	3	0	240	0
28	65	119	64	57	61	4	0	240	0
29	31	116	64	60	62	19	0	240	0
30	22	110	79	51	63	25	0	240	0
31	54	109	86	45	64	48	0	240	0
32	13	97	91	52	65	52	0	240	0
33	49	97	80	63	66	58	0	240	0
